# Multifocal Warthin's Tumor: An Uncommon Presentation of Bilateral Cervical Lymphadenopathy

**DOI:** 10.1155/2018/3791825

**Published:** 2018-09-03

**Authors:** Ryan A. Rimmer, Elizabeth E. Cottrill

**Affiliations:** Thomas Jefferson University Hospital, Department of Otolaryngology—Head & Neck Surgery, Philadelphia, PA, USA

## Abstract

Warthin's tumor is the second most common benign lesion of the parotid gland. It is most commonly encountered in male smokers in the fifth to seventh decades of life. Uniquely, among benign lesions of the parotid gland, it can be seen bilaterally in 7–10% of cases. Very rarely, Warthin's tumor can also mimic malignant or metastatic disease by presenting within cervical lymph nodes. We present a rare case of a 71-year old male smoker with bilateral parotid lesions in addition to progressively enlarging cervical and mediastinal lymphadenopathy. Excisional biopsy of a cervical lymph node ultimately revealed Warthin's tumor.

## 1. Introduction

Warthin's tumor (WT), also known as papillary cystadenoma lymphomatomsum, is the second most common benign salivary gland neoplasm representing between 6 and 10% of cases [1, 2]. While it was originally described by Hildebrand in 1895, Dr. Aldred Scott Warthin described additional cases in 1929 and the disease was ultimately renamed after him [3, 4]. It has a predominance for males with a mean age between the fifth and seventh decades. There is a strong association with smoking, but other proposed risk factors include ionizing radiation, viruses, and autoimmune disease [5].

While most commonly occurring within the parotid gland, WT can also be found more rarely in “extra-parotid” locations such as cervical lymph nodes or other salivary tissue [6]. Furthermore, these lesions can be unilateral, bilateral, or multifocal and may occur synchronously or metachronously with greater frequency than other benign salivary lesions [5]. Malignant transformation is exceedingly rare, but can take the form of either lymphomas or carcinomas [7]. We present a rare case of synchronous multifocal Warthin's tumor presenting as bilateral parotid lesions and cervical lymphadenopathy.

## 2. Case Report

A 71-year-old male presented to an outside institution in 2015 for evaluation of slowly enlarging bilateral cervical lymphadenopathy, parotid swelling, and night sweats. Medical history was significant for melanoma removed from the bridge of his nose in 2015, and active smoking for over 40 years.

During his initial workup at the OSH, computed tomography (CT) imaging showed multiple enlarged bilateral parotid lesions, scattered enlarged level I and II nodes, and upper mediastinal lymph nodes. He underwent ultrasound-guided fine needle aspiration (FNA) and core needle biopsy of a large left neck lymph node measuring 4.0 × 1.9 cm. Cytology was consistent with WT. Additionally, a level IA neck dissection was performed. Pathology revealed WT and no evidence of malignancy or lymphoma. Six-month follow-up imaging showed stable appearance of the bilateral parotid masses and cervical lymph nodes; however, there was interval enlargement of right upper mediastinal paratracheal lymph nodes.

**Figure 1:**
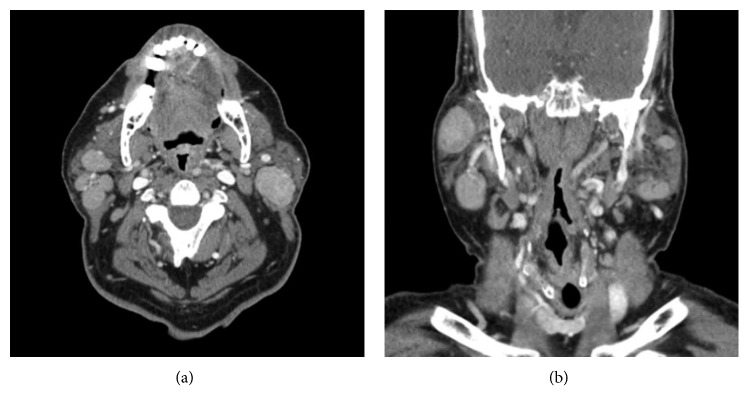
Computed tomography (CT) with contrast in axial and coronal planes showing bilateral parotid lesions and cervical lymphadenopathy.

He relocated in September 2016 and transitioned care to our institution. Thoracic surgery performed endoscopic bronchial ultrasound and transbronchial biopsies of the right paratracheal node. Cytopathology revealed absence of malignant cells. Flow cytometry was negative for lymphoma. He elected for close surveillance with serial imaging in lieu of mediastinoscopy. Six-month follow up imaging showed stable size of the mediastinal lymph nodes and slight enlargement of a left neck lymph node (Figure 1) and he was subsequently referred to the Department of Otolaryngology.

**Figure 2:**
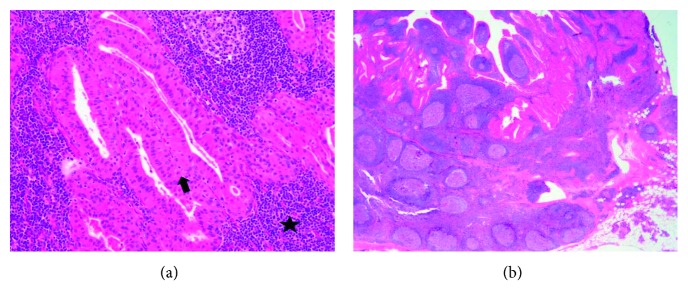
Hematoxylin and eosin stains of pathologic specimen (higher magnification on left). Note dense lymphoid stroma (star) with double layer of epithelial cells (arrow) and cystic spaces.

Physical examination revealed bilateral enlarged parotid glands and bulky cervical lymphadenopathy slightly larger on the right. Facial nerve function was intact bilaterally. The patient was counseled on smoking cessation. He was discussed at our multidisciplinary tumor board. Given the progression of cervical disease, he underwent level II lymph node dissection with removal of a large nodal conglomerate and culture of cystic contents. Histopathology once again confirmed WT (Figure 2). Culture and lymphoma workup were negative. In follow-up, he had quit smoking and elected to continue close observation.

## 3. Discussion

Unlike other benign salivary masses, WT has unique behavior in that it may occur bilaterally and outside of the major salivary glands. The pathogenesis of “extra-parotid” WT remains controversial. One hypothesis proposes that the tumor originates from heterotopic salivary parenchyma or ductal inclusions in the intra- and peri-glandular lymph nodes during the embryonic development of the parotid gland [5]. Late encapsulation of the parotid in the embryo allows the intermingling of undifferentiated lymphoid stroma with branching salivary ducts. After encapsulation, salivary ducts and acini may be trapped heterotopically within lymph nodes [8]. Heterotopic salivary gland tissue in the neck may arise sporadically via failed closure of the pre-cervical sinus of His within the branchial apparatus [9]. Genetic and environmental factors may then impact these inclusions giving rise to Warthin's tumor.

Other theories using immunohistochemistry propose that WT results from inflammation directed at an unknown antigen. The antigen may be epithelial cells infected by a virus or mutated by tobacco smoke leading to immune response. Alternatively, the inflammation may be an autoimmune response to its own duct cells [10]. Some propose that the tumor is an adenoma with concomitant lymphocytic infiltration; however, this theory has less support given that many classify WT as a “tumor-like lesion” since the epithelial and lymphoid components have been shown to be polyclonal in origin [11, 12].

Tobacco smoking is closely associated with WT. Smoking may trigger ductal hyperplasia and oncocytic metaplasia in preexisting lymph nodes [13]. This transformation occurs with advanced age, which may help to explain the observed age range. Mitochondria-rich oncocytic cells may show structural abnormalities and reduced metabolic function. Smoking may potentiate this damage due to development of reactive oxygen species. Studies have detected high rates of deleted mitochondrial DNA in oncocytic cells of WT [14].

Histologically, WT tissue displays epithelial and lymphoid components. Glandular and cystic structures feature a bilayered epithelial lining composed of inner columnar eosinophilic or oncocytic cells surrounded by smaller basal cells. The lymphoid stroma may display varying degrees of reactivity. A sharply demarcated thin capsule surrounds Warthin's tumor and there may be extensive fibrosis at the periphery [10].

On CT imaging, WT displays oval or round lesions with uniform or heterogeneous density, a clear edge, and no calcifications [15]. These characteristics are consistent with the findings in our case. On magnetic resonance imaging (MRI), T1 shows low to intermediate signal, variable intensity on T2, and lack of enhancement with contrast [16]. Using MR diffusion weighted imaging (DWI), WT has a lower apparent diffusion coefficient (ADC) than pleomorphic adenoma and a comparable ADC to malignant salivary tumors [17]. A low ADC indicates decreased diffusion of water molecules in tissue, suggesting more cellular tissue. Generally, malignant tumors tend to have lower ADC than benign tumors [18]. WT may be differentiated from malignant lesions based on “hot” uptake with ^99m^Tc pertechnetate scintography, which is due to the epithelial component's ability to concentrate ^99m^Tc pertechnetate but not secrete it [18, 19]. WT also exhibits intense uptake on FDG-PET/CT [20].

Patients are typically asymptomatic until identifying a mass. Lesions are most commonly slow growing and firm or fluctuant to palpation. The slow growing nature has been attributed to low levels of MMP-1/Ki-67 expression, which has been associated with reduced cell proliferation [10]. WT typically occurs in the lower pole of the superficial lobe of the parotid gland and rarely in the deep lobe (10%). It can be multicentric in 12–20% and/or bilateral in 5–14% [10]. Multifocal extra-parotid Warthin's tumor has been reported to occur in only 2% of cases [11]. Our patient suffered from bilateral superficial parotid lesions and multifocal cervical lesions. Uniquely, our patient also had mediastinal lymphadenopathy although the EBUS cytology was not diagnostic of WT. Mediastinoscopy could provide additional diagnostic tissue, but was deferred at this time.

Given the low risk of malignancy, conservative management with close observation is often a reasonable consideration. For cases in which surgical resection is sought for cosmesis, superficial parotidectomy is most commonly recommended since WT occurs predominantly in the superficial parotid lobe. Benefits of resection must be weighed against the risk of iatrogenic facial nerve injury and overall risks of anesthesia especially in the case of bilateral tumors. The multifocal nature of WT has made extracapsular dissection more prone to recurrence as residual disease may be left behind [11].

## 4. Conclusions

Warthin's tumor (papillary cystadenoma lymphomatosum) is the second most common benign lesion of the salivary glands. While most commonly occurring in the parotid gland, unlike other benign salivary masses, it has a propensity for bilaterality and may occur in various extra-parotid locations including cervical lymph nodes. There is a strong association with tobacco smoking, although the exact pathogenesis of Warthin's tumor remains a controversial topic. The risk of malignant transformation is extremely low. In smokers with cervical lymphadenopathy, the suspicion for malignancy is high, however, Warthin's tumor should remain on the differential.
